# Fetal growth according to different reference ranges in twin pregnancies with placental insufficiency

**DOI:** 10.6061/clinics/2015(12)09

**Published:** 2015-12

**Authors:** Julianny Cavalheiro Nery Nakano, Adolfo Wenjaw Liao, Maria de Lourdes Brizot, Mariana Miyadahira, Rossana Pulcineli Vieira Francisco, Marcelo Zugaib

**Affiliations:** Hospital das Clínicas da Faculdade de Medicina da Universidade de São Paulo, Departamento de Obstetrícia e Ginecologia, São Paulo/SP, Brazil.

**Keywords:** Twin Pregnancy, Fetal Growth, Abdominal Circumference, Estimated Weight, Ultrasonography, Placental Insufficiency

## Abstract

The aim of this study was to compare different fetal growth curves in twin pregnancies with severe placental insufficiency. A retrospective cross-sectional analysis of 47 twin pregnancies with absent or reverse end diastolic flow in the umbilical artery of one fetus was performed. Pregnancies with major fetal abnormalities, twin-twin transfusion or three or more fetuses were not included. The estimated fetal weight zeta-scores were calculated for both fetuses (abnormal Doppler and co-twin) according to the following criteria: Hadlock, Liao and Araújo. The abdominal circumference zeta-scores were calculated according to Hadlock, Liao, Araújo, Ong and Stirrup. The mean estimates of the zeta-score values were calculated using generalized estimating equation regression analysis.

The mean gestational age at inclusion was 27.4±4.7 weeks. The fetal sex and the interaction Doppler findings × criteria correlated significantly with the zeta-score values (*p*<0.001 for both variables). The estimated fetal weight mean zeta-scores (standard error) according to each criteria were as follows: Hadlock - abnormal Doppler: -2.98 (0.18), co-twin: -1.16 (0.15); Liao - abnormal Doppler: -2.89 (0.24), co-twin: -0.58 (0.19); and Araújo - abnormal Doppler: -3.05 (0.29), co-twin: -0.75 (0.18). Values for abdominal circumference were as follows: Hadlock - abnormal Doppler: -3.14 (0.26), co-twin: -1.13 (0.19); Liao - abnormal Doppler: -2.63 (0.27), co-twin: -0.42 (0.19); Araújo - abnormal Doppler: -2.44 (0.22), co-twin: -0.71 (0.14); Ong - abnormal Doppler: -3.36 (0.34), co-twin: -1.48 (0.23); and Stirrup AD -- -2.36 (0.14), co-twin: -1.18 (0.10).

Sex- and plurality-specific charts should be used in the evaluation of fetal growth in twin pregnancies with placental insufficiency.

## INTRODUCTION

Twin pregnancies are associated with lower mean birthweights compared with singletons. Fetal growth deviation begins after the end of the second trimester and increases progressively throughout pregnancy 1. The twin growth patterns and discrepancies between fetuses within the same pregnancy may in fact be determined by a multitude of factors, including maternal (nutritional status, uterine and hormonal environment), fetal (genetics, epigenetics and metabolic) and placental factors 2.

Despite being a simple and inexpensive method, symphysis-fundal height tape measurements do not allow an adequate evaluation of growth for each fetus in a multiple pregnancy. Moreover, this measurement is of value only when both babies are small 3.

Therefore, the assessment of twin fetal growth is currently based essentially on serial ultrasound scans. However, most centers still use singleton reference charts to evaluate these pregnancies. This inevitably leads to the frequent misdiagnosis of fetal growth restriction, increased parental anxiety, increased financial costs and unnecessary iatrogenic deliveries.

We have previously published a prospective longitudinal study describing reference ranges for ultrasound fetal biometry and growth in uncomplicated twin pregnancies 4, and additional reference ranges are also available from other twin studies 5,7. The aim of the present study was to evaluate these growth curves in twin pregnancies at an increased risk of an adverse perinatal outcome due to severe placental insufficiency.

## MATERIAL AND METHODS

This was a retrospective cross-sectional study involving twin pregnancies with placental insufficiency seen at the Department of Obstetrics and Gynecology, São Paulo University Medical School Hospital, between 2005 and 2013.

A computer database search was performed to identify all twin pregnancies with absent or reverse end diastolic flow in the umbilical artery of one fetus (AD) and normal Doppler findings in the co-twin (CT). The perinatal outcomes and clinical data were retrieved from the hospital notes. We excluded pregnancies that had an unknown outcome, that were complicated by twin-to-twin transfusion syndrome or that exhibited a major fetal abnormality.

The gestational age was calculated from the first day of the last menstrual period (LMP) and confirmed by a crown-rump length measurement during the 1^st^ trimester or by an estimate based on multiple ultrasound parameters (biparietal diameter, head circumference, abdominal circumference and femur length) of the largest fetus during the 2^nd^ trimester. When the LMP was uncertain or unknown, or when there was a discrepancy between the LMP and ultrasound dates, the gestational age was determined based on the earliest ultrasound findings. The pregnancy chorionicity was determined by ultrasound and confirmed by placental histological examination after birth.

The fetal abdominal circumference (AC) measurement and estimated weight (EFW, according to the mathematical formula published by Hadlock et al.), at the moment of the diagnosis of absent or reverse end-diastolic flow in the umbilical artery, were retrieved for both twins. The zeta-score values were calculated as follows: (observed value - expected mean value for gestation)/standard deviation according to different reference ranges published in the literature (Hadlock et al. 8, Liao et al. 4, Araujo et al. 6, Ong et al. 5 and Stirrup et al. 7).

A generalized estimating equation regression analysis was used to examine the association of zeta-score values with maternal age, pregnancy chorionicity, fetal sex, gestational age at the ultrasound examination, Doppler findings and reference criteria. The analysis was performed using the Statistical Package for Social Science (version 20) and the significance level was set as 0.05.

## ETHICS

The study protocol was registered and approved by the Institutional Ethics Review Board (CAPPesq: 70648).

## RESULTS

The database search identified 78 multiple pregnancies with absent or reverse end-diastolic flow in the umbilical artery; both fetuses were affected in one pregnancy, 4 were triplets, 8 had twin-to-twin transfusion syndrome, 9 had a major fetal abnormality and the outcome was unknown in another 9 cases. Therefore, the final data analysis was based on 47 twin pregnancies.

**Figure 1:**
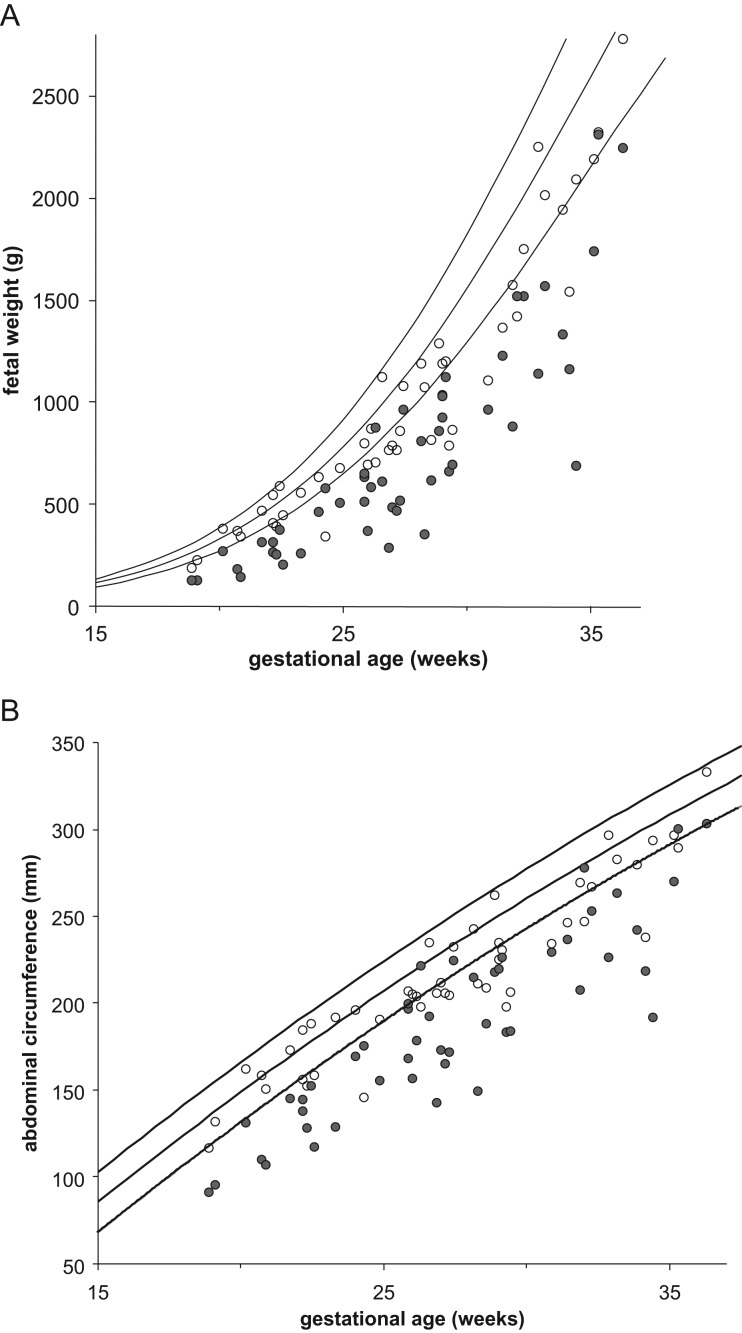
Scatterplots of estimated fetal weight (A) and abdominal circumference measurements (B) of 47 twin pregnancies with placental insufficiency plotted against singleton reference ranges. Closed circles: fetuses with absent or reverse end-diastolic flow in the umbilical artery; open circles: co-twin with normal Doppler.

The maternal characteristics and pregnancy information are summarized in Table 1. Tables 2 and 3 present the estimated fetal weight and abdominal circumference measurement distribution when absent or reverse end-diastolic flow in the umbilical artery was first detected in this population (27.4±4.7 weeks). Figure 1 presents scatterplots of these values against singleton reference ranges.

The generalized estimating equation regression analysis revealed that the fetal sex and the interaction Doppler findings x criteria correlated significantly with the zeta-score values (*p*<0.001 for both variables).

**Figure 2:**
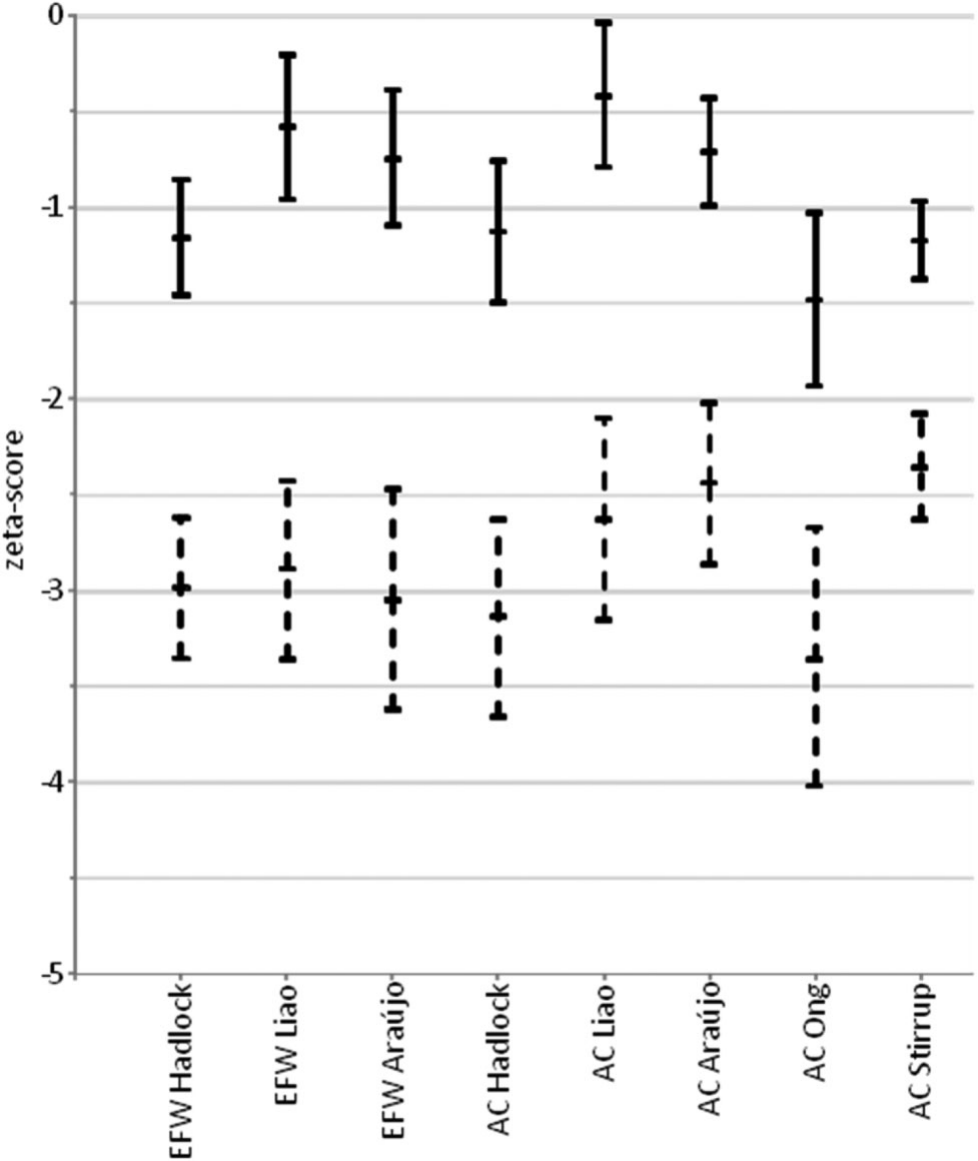
Mean estimate and 95% confidence interval for estimated fetal weight (EFW) and abdominal circumference (AC) zeta-score values according to different reference ranges in 47 twin pregnancies with placental insufficiency. Dashed lines: absent or reverse end-diastolic flow in the umbilical artery group; continuous lines: normal umbilical artery Doppler group.

The mean zeta-score estimate was significantly greater in male fetuses (−1.52, 95% confidence interval CI: -1.68 to -1.36) compared with females (−2.26, 95%CI: -2.40 to -2.12, mean difference: 0.74±0.11, *p*<0.001). Table 4 summarizes the EFW and AC zeta-score estimates according to umbilical artery Doppler findings and reference ranges (Figure 2).

## DISCUSSION

The present study demonstrates very significant differences in fetal growth deviation between fetuses with an abnormal umbilical artery Doppler and their respective co-twins. Approximately 80% and 30% of fetuses with abnormal Doppler findings and their co-twins, respectively, were classified below the 10^th^ percentile.

Nevertheless, it is important to emphasize that our inclusion criteria were very stringent due to the retrospective nature of the study. Absent end-diastolic flow certainly represents the most extreme degree of placental insufficiency and further longitudinal studies will help clarify when differences in fetal growth become apparent before the absence of diastolic flow occurs in the umbilical artery.

Asymmetrical fetal growth is one of the characteristics that helps to differentiate constitutionally small fetuses from abnormal fetal growth restriction. Therefore, one would expect a greater reduction in the abdominal circumference zeta-scores compared with the estimated fetal weight. However, this is not apparent in our data (Figure 1), and a possible explanation is that early severe placental function compromise affects fetal growth in a global manner.

Generalized estimating equation (GEE) analysis is a useful tool to adjust for repeat measurements and related data 9. Because fetuses within a twin pregnancy cannot be considered independent subjects, GEE analysis was used to overcome a certain degree of correlation that is likely to exist among individual responses within the same pregnancy.

Approximately 25-30% of the fetuses with normal flow in the umbilical artery were below the 10th percentile of a reference curve that was prospectively constructed with uncomplicated twin pregnancies within the same department 4. This finding suggests that in pregnancies with severe placental insufficiency affecting one fetus, some degree of growth compromise occurs in the co-twin, possibly mediated by an abnormal production of placental angiogenic factors.

Moreover, the GEE technique allows proper adjustments for pregnancy chorionicity, fetal sex, gestational age and the fact that, in the present study, a single measurement (EFW or AC value) was converted into different zeta-score values according to each criteria.

Although some studies 6,7 have demonstrated differences in fetal growth when comparing monochorionic and dichorionic twins, our present data do not support the need for chorionicity adjustments when interpreting fetal growth deviation in pregnancies with severe placental insufficiency.

Interestingly, fetal sex was the only additional parameter that correlated significantly with the zeta-score values, which confirms the need to interpret growth according to sex-specific charts, as has been discussed by Fenton & Kim 10.

It is a very common practice to use the reference ranges published by Hadlock et al. 8 for singleton pregnancies when evaluating a twin pregnancy. However, our data indicate that this approach yields an excess of approximately 10% in the number of cases classified below the 10th percentile.

Although the reference values published by Ong et al. were based on a population of twin pregnancies, these criteria were associated with the lowest estimates of the mean zeta-score. This finding could be attributed to methodological issues in that study, which was a retrospective review of data collected over 13 years by a heterogeneous group of operators including radiographers, midwives and obstetricians 5.

In conclusion, sex- and plurality-specific charts should be used in the evaluation of fetal growth in twin pregnancies with placental insufficiency.

## AUTHOR CONTRIBUTIONS

Nakano JC collected the data and drafted the manuscript. Liao AW collected the data, performed the statistical analysis and drafted the manuscript. Brizot ML collected the data and revised the manuscript. Miyadahira M collected the data. Francisco RP revised the manuscript. Zugaib M revised the manuscript and contributed to discussions.

## Figures and Tables

**Table 1:** Maternal characteristics and pregnancy information in 47 twin pregnancies with absent or reverse end-diastolic flow in the umbilical artery of one fetus.

	Mean ± standard deviation / n (%)
Maternal age, years	27.8±7.4
First pregnancy	24 (51)
Chorionicity	
Monochorionic	29 (61.7)
Dichorionic	15 (31.9)
Not confirmed by histological examination	3 (6.4)
Intrauterine death	
Abnormal Doppler fetus	10 (21.3)
Normal Doppler co-twin	1 (2.1)
Gestational age at delivery, weeks	32.9±2.9
Delivery <32 weeks	17 (36.2)
Delivery <34 weeks	29 (61.7)
Mode of delivery	
Vaginal	5 (10.6)
Cesarean	42 (89.4)
Birthweight, grams	
Abnormal Doppler fetus	1075±470
Normal Doppler co-twin	1750±544
Sex	
Female	48 (51.1)
Male	43 (45.7)
Not determined	3 (3.2)

**Table 2:** Estimated fetal weight distribution in 47 twin pregnancies with placental insufficiency according to different reference ranges. AD: fetuses with absent or reverse end-diastolic flow in the umbilical artery; CT: normal umbilical artery Doppler co-twin. The relative values are given in brackets.

**Percentile**	**Hadlock et al. (7)**		**Liao et al. (3)**		**Araújo et al. (5)**	
	**AD**	**CT**	**AD**	**CT**	**AD**	**CT**
<5	40 (85.1)	17 (36.2)	36 (76.6)	10 (21.3)	32 (72.7)	14 (31.8)
<10	44 (93.6)	20 (42.6)	39 (83.0)	16 (30.0)	36 (81.8)	16 (36.4)
10 to 50	3 (6.4)	21 (44.7)	6 (12.8)	15 (31.9)	8 (18.2)	16 (36.4)
>50	-	6 (12.8)	2 (4.2)	16 (34.0)	-	12 (27.3)
Total	47 (100)	47 (100)	47 (100)	47 (100)	44 (100)	44 (100)

**Table 3:** Fetal abdominal circumference distribution in 47 twin pregnancies with placental insufficiency according to different reference ranges. AD: fetuses with absent or reverse end-diastolic flow in the umbilical artery; CT: normal umbilical artery Doppler co-twin. The relative values are given in brackets.

**Percentile**	**Hadlock et al. (7)**		**Liao et al. (3)**		**Araújo et al. (5)**		**Ong et al. (4)**		**Stirrup et al. (6)**	
	**AD**	**CT**	**AD**	**CT**	**AD**	**CT**	**AD**	**CT**	**AD**	**CT**
<5	41 (87.3)	14 (29.8)	32 (68.1)	7 (14.9)	30 (68.2)	8 (18.2)	30 (85.7)	14 (40)	36 (81.8)	9 (20.5)
<10	42 (89.4)	21 (44.7)	38 (80.9)	11 (23.4)	33 (75)	14 (31.8)	-	18 (51.4)	40 (90.9)	19 (43.2)
10 to 50	4 (8.5)	14 (29.8)	4 (8.5)	19 (40.4)	9 (20.5)	17 (38.6)	5 (14.3)	12 (34.3)	4 (9.1)	24 (54.5)
>50	1 (2.1)	12 (25.5)	5 (10.6)	17 (36.2)	2 (4.5)	13 (29.6)	-	5 (14.3)	-	1 (2.3)
Total	47 (100)	47 (100)	47 (100)	47 (100)	44 (100)	44 (100)	35 (100)	35 (100)	44 (100)	44 (100)

**Table 4:** Estimated fetal weight and abdominal circumference zeta-score values according to different reference ranges in 47 twin pregnancies with absent or reverse end-diastolic flow in the umbilical artery of one fetus. The values are given as the average ± standard error of the estimate (95% confidence interval).

**Criteria**	**Abnormal Doppler twin**	**Normal Doppler co-twin**
**Estimated fetal weight**		
Hadlock et al. (7)*	−2.98±0.18 (−3.35 to -2.62)	−1.16±0.15 (−1.46 to -0.86)
Liao et al. (3)*	−2.89±0.24 (−3.36 to -2.43)	−0.58±0.19 (−0.96 to -0.21)
Araújo et al. (5)*	−3.05±0.29 (−3.62 to -2.47)	−0.75±0.18 (−1.10 to -0.39)
**Abdominal circumference**		
Hadlock et al. (7)*	−3.14±0.26 (−3.66 to -2.63)	−1.13±0.19 (−1.50 to -0.76)
Liao et al. (3)*	−2.63±0.27 (−3.15 to -2.10)	−0.42±0.19 (−0.79 to -0.04)
Araújo et al. (5)*	−2.44±0.22 (−2.86 to -2.02)	−0.71±0.14 (−0.99 to -0.43)
Ong et al. (4)*	−3.36±0.34 (−4.02 to -2.67)	−1.48±0.23 (−1.93 to -1.03)
Stirrup et al. (6)*	−2.36±0.14 (−2.63 to -2.08)	−1.18±0.10 (−1.38 to -0.97)

* *p*<0.001
